# The Impact of Psoriasis and Metabolic Syndrome on the Systemic Inflammation and Oxidative Damage to Nucleic Acids

**DOI:** 10.1155/2020/7352637

**Published:** 2020-05-20

**Authors:** Drahomira Holmannova, Lenka Borska, Ctirad Andrys, Pavel Borsky, Jan Kremlacek, Kvetoslava Hamakova, Vit Rehacek, Andrea Malkova, Tereza Svadlakova, Vladimir Palicka, Jan Krejsek, Zdenek Fiala

**Affiliations:** ^1^Institute of Hygiene and Preventive Medicine, Faculty of Medicine in Hradec Kralove, Charles University, Czech Republic; ^2^Institute of Pathological Physiology, Faculty of Medicine in Hradec Kralove, Charles University, Czech Republic; ^3^Institute of Clinical Immunology and Allergology, University Hospital and Faculty of Medicine in Hradec Kralove, Charles University, Czech Republic; ^4^Clinic of Dermatology and Venereology, University Hospital Hradec Kralove, Czech Republic; ^5^Transfusion Center, University Hospital, Hradec Kralove 500 03, Czech Republic; ^6^Institute of Clinical Biochemistry and Diagnostics, University Hospital Hradec Kralove and Faculty of Medicine in Hradec Kralove, Charles University, Czech Republic

## Abstract

**Background:**

Psoriasis is a chronic systemic inflammatory disease associated with a wide range of comorbidities, including metabolic syndrome (MetS). Serum calprotectin, ANGPTL8, and oxidative damage to nucleic acids might be associated with both diseases. The presented study describes the influence of psoriasis and MetS on the serum levels of markers of systemic inflammation (calprotectin and ANGPTL8) and markers of oxidative damage to nucleic acids. The applicability of serum levels of calprotectin and ANGPTL8 for monitoring of the activity of psoriasis (diagnostic markers) is also evaluated.

**Methods:**

Clinical examination (PASI score, MetS), enzyme-linked immunosorbent assay (ELISA), and Enzyme Immunoassay (EIA). Serum calprotectin, ANGPTL8, 8-hydroxy-2′-deoxyguanosine, 8-hydroxyguanosine, and 8-hydroxyguanine. *Results and Conclusions*. The psoriasis significantly increased the serum level of calprotectin and the serum level of oxidative damage to nucleic acids, however not the serum level of ANGPTL8. The presence of MetS did not significantly affect the serum levels of calprotectin, ANGPTL8, and oxidative damage to nucleic acids in either psoriasis patients or controls. It seems that the serum level of calprotectin (but not the serum level of ANGPTL8) could be used as a biomarker for monitoring the activity of psoriasis.

## 1. Introduction

Inflammation is a precisely controlled, well-regulated response of the immune system to diverse stimuli, which provides protection and defense against both external and internal danger (such as pathogens, toxins, chemicals, cancer, and damaged cells) and promotes regeneration of damaged tissue. A wide range of factors can disrupt immune system functions (immune system homeostasis, self-tolerance, inhibitory/anti-inflammatory mechanisms, termination of inflammation, proresolving, and regenerative processes) and thus transform initially healing process into a destructive immune system response.

Psoriasis, metabolic syndrome (MetS), and oxidative stress are accompanied by low-grade chronic inflammation [1, 2]. Psoriasis is a chronic systemic genetically linked inflammatory (immune-mediated) hyperproliferative condition with a diverse array of clinical manifestations [3, 4]. Psoriasis preferentially affects the skin and also the nails, joints, mucous membranes, etc. The pathogenesis of psoriasis is still not fully understood. Multiple factors are known to be implicated into the pathogenesis of psoriasis: genetic predisposition (polymorphisms in genes controlling different immunological signaling pathways and processes), abnormal individual reactivity, epigenetic factors, oxidative stress, or skin microbiome alteration [5–9]. These factors alter skin barrier functions; cause defects in differentiation and proliferation of keratinocytes; increase the skin infiltration by immune cells (T cells, macrophages, neutrophils, and dendritic cells); intensify the production of IL-23, IL-22, IL-17A, TNF*α*, INF*γ*, and IL-12; and drive the assembly of inflammasomes, and thus the inflammation is triggered and maintained [10, 11].

Oxidative stress, reactive oxygen and nitrogen species, is a key player in the pathogenesis of many human diseases. A wide range of environmental factors (toxins, chemicals, and UV radiation) that interact with the skin and inflammatory reactions in the organisms enhance oxidative stress which then damages cells, their membranes and DNA/RNA, and leads to the release of DAMPs (damage/danger-associated molecular patterns) which include alarmins such as IL1*α*, HMGB1, and calprotectin, as well as boosts the immune-inflammatory response. Studies have shown that the treatment with antioxidant ameliorates symptoms of inflammatory diseases and reduces their progression (e.g., psoriasis, rheumatoid arthritis, and neurodegenerative or cardiovascular diseases) and decreases the level of calprotectin [12–14].

Calprotectin is a heterocomplex of two calcium-binding molecules S100A8 and S100A9. These alarmins belong to the S100 protein family and are released in heterodimeric form from immune cells in the response to the environmental triggers, oxidative stress, cellular damage, bacterial infections, and inflammation. The main source of calprotectin is neutrophils, monocytes, and macrophages [15]. Calprotectin can serve as a noninvasive biomarker for inflammatory diseases (for example, IBD, rheumatoid arthritis, infections, cardiovascular diseases, MetS, and bronchiolitis obliterans). It can help diagnose inflammation and predict relapse in patients in clinical remission [16–19].

There is also a link between inflammation and MetS and thus psoriasis as well. The MetS is associated with overweight or obesity. Adipose tissue is an active endocrine organ which produces adipokines and proinflammatory cytokines that attract immune system cells, mainly monocytes, and support differentiation of infiltrating macrophages into M1 proinflammatory subset; therefore, the presence of the MetS might negatively regulate the course psoriasis and vice versa [20–23]. The MetS, as well as dyslipidemia, insulin resistance, and fatty liver disease, is characterized by an increased expression of angiopoietin-like 8 (ANGPTL8; betatrophin). ANGPTL8 is a liver and adipose tissue-specific glycoprotein with ability to regulate both glucose and lipid metabolism by inhibition of lipoprotein lipase. ANGPTL8 is rhythmically expressed, but the level of expression depends on stimuli such as insulin and calorie intake [24–26].

The study describes the influence of psoriasis and MetS on the serum indicators of systemic inflammation (calprotectin and ANGPTL8) and on the serum indicators of oxidative damage to nucleic acids. The paper also evaluates the applicability of serum levels of calprotectin and ANGPTL8 for monitoring of the activity of psoriasis (diagnostic markers).

## 2. Methods

### 2.1. Study Groups

The experimental group consisted of 44 patients with psoriasis. Patients were examined at the Clinic of Dermal and Venereal Disease, Charles University Hospital in Hradec Kralove. The control group included 80 healthy blood donors from the Department of Transfusion Medicine, Charles University Hospital in Hradec Kralove. All subjects signed the informed consent before participating in the study. The persons with any inflammatory diseases, pregnancy, and those using nonsteroidal or anti-inflammatory medications were excluded. Patients had no treatment of psoriasis in three months before the enrollment of the study.

The study was conducted in accordance with the Declaration of Helsinki, and the protocol was approved by the Ethics Committee of the Faculty Hospital in Hradec Kralove, Czech Republic (project identification code PROGRES Q40-09 and Q40-10, reference number 201705 I83P, date 2 May 2017).

### 2.2. Disease Status Determination: PASI Score

The severity of the disease was assessed using a standardized clinical evaluation—Psoriasis Area Severity Index (PASI), which is calculated based on erythema, desquamation, and skin infiltration [27].

### 2.3. Metabolic Syndrome (MetS)

We followed the methods of Borska et al. We evaluated the presence of MetS in observed subjects according to the criteria of the National Cholesterol Education Program Adult Treatment Panel (NCE/ATPIII). The diagnosis of MetS was declared when three of the five listed criteria were present: (1) increased waist circumference or abdominal obesity (≥102 cm for men; ≥88 cm for women), (2) glucose intolerance (fasting glucose ≥ 5.6 mmol/L or known treatment of diabetes), (3) elevated level of triglycerides (TAG) ≥ 1.7 mmol/L, (4) reduced level of high-density lipoproteins (HDL cholesterol), and (5) elevated blood pressure (>130/85 mmHg) [28, 29].

### 2.4. Blood Sample Collection

Peripheral blood samples were collected from the cubital vein in both groups by BD Vacutainer sampling tubes. Blood serum was isolated by centrifugation, and the samples were stored at −70°C. Repeated thawing and freezing were avoided.

### 2.5. Analysis of Calprotectin

Concentrations of calprotectin were analyzed in the serum samples with enzyme-linked immunosorbent assay (ELISA) using the Human S100A8/A9 (calprotectin) ELISA kit (BioVendor, Czech Republic) according to the manufacturer's instructions. Samples were 200-fold diluted. The sensitivity of the kit was 0.22 ng/mL. The absorbance values were read at 450 nm on a Multiskan RC ELISA reader (Thermo Fisher Scientific, Waltham, MA, USA).

### 2.6. Analysis of ANGPTL8

Serum levels of ANGPTL8 were determined by using the ELISA kit (enzyme-linked immunosorbent assay kit) for ANGPTL8 (Cloud-Clone Corp., TX, USA) according to the manufacturer's instructions. Detection limit of the kit was 0.056 ng/mL. Samples were 30-fold diluted. The absorbance values were read at 450 nm on a Multiskan RC ELISA reader (Thermo Fisher Scientific, Waltham, MA, USA).

### 2.7. Analysis of Oxidative Damage to Nucleic Acids

The EIA Kit (Enzyme Immunoassay, Cayman Chemical Company, Ann Arbor, MI, USA) was used to measure the level of oxidative damage to nucleic acids (DNA/RNA damage). The damage was shown as the sum of three oxidized guanine species in serum: 8-hydroxy-2′-deoxyguanosine released from DNA, 8-hydroxyguanosine from RNA, and 8-hydroxyguanine from DNA or RNA. We measured the level of DNA/RNA damage in pg of all guanine species per mL of serum. The detection limit was 33 pg/mL of serum. We followed the methods of Borska et al. [28].

### 2.8. Statistical Analysis

The data were statistically processed by the R software version 3.6.1 “nortest,” “compute.es,” and “ggplot2.” Based on normality distribution evaluation (the Anderson–Darling test), parametric or nonparametric tests were used. The relationship between parameters was evaluated either by Pearson's or by Spearman's correlation test. Differences among groups were assessed using Student's *t* or Wilcoxon rank-sum test. The null hypothesis was rejected when the probability level (*p*) reached below 0.05 (the alpha level) [30–32].

## 3. Results

### 3.1. Study Groups

**Figure 1:**
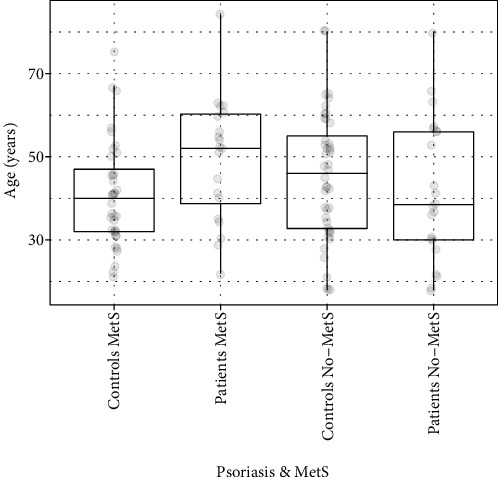
Age characteristics of monitored groups. Legend: data are graphically displayed as box blots (the minimum value is represented by points below the box, 1^st^ line (bottom) of the box represents first quartile (Q1), 2^nd^ (middle of the box) median, 3^rd^ (top of the box) third quartile (3Q), points above the box represent the maximum value).

A total of 124 subjects were examined: 44 patients with psoriasis (22 patients without metabolic syndrome = patients No-MetS and 20 patients with metabolic syndrome = patients MetS) and 80 healthy controls (44 controls without metabolic syndrome = controls No-MetS and 36 controls with metabolic syndrome = controls MetS). The age distribution did not differ significantly between patients and controls (average age: 45.4 vs. 43.6; min–max age: 18−84 vs. 18−80; Figure 1).

**Figure 2:**
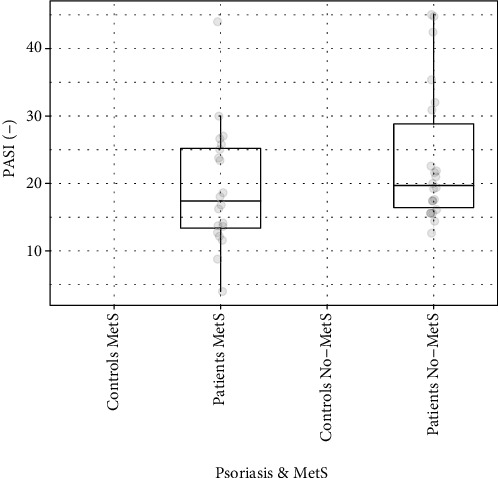
The PASI score in patients with and without MetS.

We found that PASI scores of patients MetS were 17.4/19.3 (median/mean) and patients No-MetS 19.7/23.6 (Figure 2). No significant difference in the PASI score was observed between patients MetS and patients No-MetS.

### 3.2. Analysis of Calprotectin

**Figure 3:**
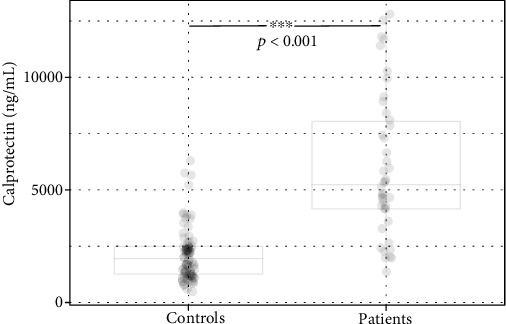
The levels of calprotectin in controls and patients.

**Figure 4:**
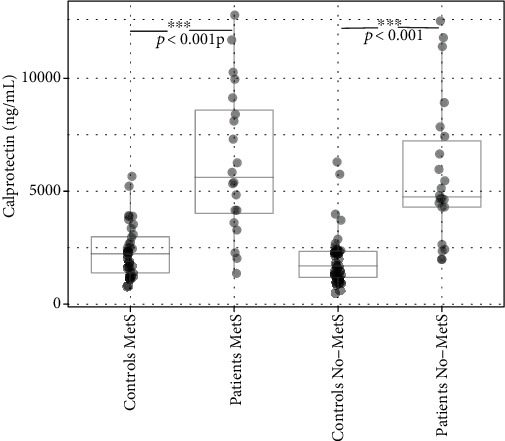
The levels of calprotectin in controls and patients with and without MetS.

The serum level of calprotectin was significantly elevated in patients compared to the controls regardless of the presence of MetS (*p* < 0.001; Table 1, Figures 3 and 4). In both, the patient and control groups, the presence of MetS resulted in an insignificant increase in calprotectin levels (Table 2, Figure 4).

### 3.3. Analysis of ANGPTL8

No significant differences in the serum level of ANGPTL8 were observed between patients and controls, even among subgroups (Table 1). Neither in the patient group nor in the control group has the presence of MetS significantly influence the ANGPTL8 level compared to those without MetS (Table 2).

### 3.4. Analysis of Oxidative Damage to Nucleic Acids

**Figure 5:**
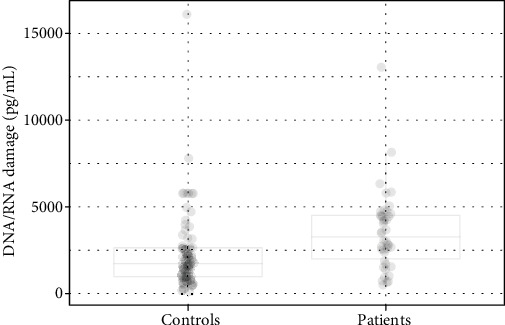
The levels of oxidative damage to nucleic acids in controls and patients.

**Figure 6:**
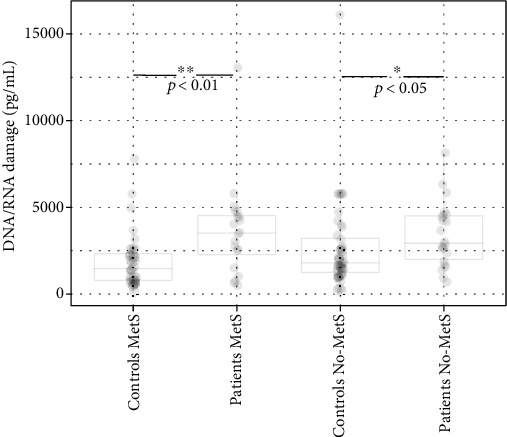
The levels of oxidative damage to nucleic acids in controls and patients with and without MetS.

The serum level of the oxidative damage to nucleic acids was significantly elevated in patients compared to controls regardless of the presence of MetS (*p* < 0.001; Table 1, Figures 5 and 6). Neither in the patient group nor in the control group has the presence of MetS significantly influence the oxidative damage to nucleic acids compared to those without MetS (Table 2, Figure 6).

### 3.5. Relationships among Measured Parameters

**Figure 7:**
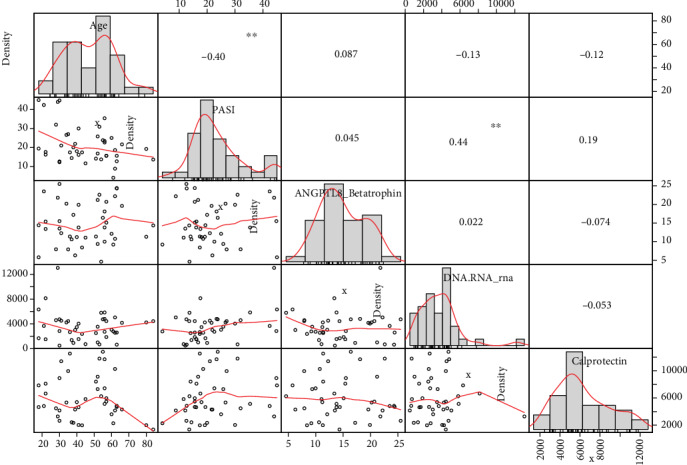
Relationships among measured parameters (Spearman rank correlation).

The relations between the age, PASI score, ANGPTL8, oxidative damage to nucleic acids, and calprotectin were investigated in patients. We found negative correlation between the age and PASI score (*r* = −0.40; *p* < 0.01) and positive correlation between the PASI score and oxidative damage to nucleic acids (0.44; *p* < 0.01) (Figure 7).

## 4. Discussion

Psoriasis is a chronic systemic multifactorial inflammatory disease with genetic predisposition which is accompanied with the activation of the immune system cells, resident skin cells, increased production of cytokines, chemokines, and other soluble substrates; some of them could serve as a screening indicators for psoriasis (for diagnostics, prognostics, or monitoring the treatment efficacy), for oxidative stress, and for MetS.

Previous studies have shown that serum levels of calprotectin and oxidative damage to nucleic acids are increased in patients with psoriasis. Also, in our study, the levels of calprotectin were significantly higher in patients with psoriasis compared to control. The presence of MetS led to an increase in calprotectin levels in patients and controls; however, this increase did not reach the level of statistical significance. Other authors obtained similar results as we did [33–35].

Calprotectin can be considered as a promising biomarker of inflammation, psoriasis, and its complication, such as arthritis. Zaki et al. as well as D'Amico et al. confirmed that biological therapy decreased both inflammation and the level of calprotectin [34, 35]. Zaki et al., Hamza et al., and Greco et al. documented that the levels of calprotectin significantly correlated with the PASI score; however, in our study, the correlation between PASI score and calprotectin was not statistically significant [33, 34].

As we mentioned previously, the level of calprotectin was increased in our patients with psoriasis, especially in patients with metabolic syndrome. Unsurprisingly, the same pattern of calprotectin secretion was documented in healthy controls; increased levels were in control with metabolic syndrome. Psoriasis is undoubtedly associated with inflammation and metabolic syndrome which is also inflammatory condition; thus, both psoriasis and metabolic syndrome might be accompanied by the elevated level of calprotectin. Pedersen et al. also proved that patients with diabetes mellitus type II with clinical complication such as cardiovascular disease and obesity had higher concentration of plasma calprotectin than the general population; they also described a positive correlation between the level of calprotectin BMI, triglycerides, hs-CRP, insulin, and negative correlation with HDL cholesterol, etc. [18]. Metabolic syndrome includes, besides other disorders, obesity and atherosclerosis. Obesity has been shown to promote expansion of Th17 cells and IL-17 production in adipose tissue; atherosclerosis is defined as a chronic inflammatory process with endothelial dysfunction, deposits of lipids, and macrophages in the arterial walls; moreover, it has been reported that Th17 and IL-17A are involved in the progression of atherosclerosis. Th17 and IL-17 are important players in pathogenesis of psoriasis, one of the many proinflammatory signals linking atherosclerosis (obesity and metabolic syndrome) and psoriasis [36, 37].

Pirowska et al. assessed the levels of proinflammatory cytokines IL-23, IL-17, etc. in serum of patients with psoriasis and psoriatic arthritis in order to establish the correlation between cytokines and PASI score and risk of obesity and metabolic syndrome. Psoriatic patients with metabolic syndrome had a higher level of serum IL-17 and IL-23 than patients without metabolic syndrome [38]. But we have to take into consideration that there are other factors involved in the pathogenesis of psoriasis and metabolic syndrome. These processes are much more complex and influenced by many endogenous and exogenous factors, e.g., oxidative stress. Oxidative stress is a condition that refers to an imbalance between the production of oxygen/nitrogen reactive species and the ability of an organism to detoxify them. Inflammation and oxidative stress are closely linked and implicated in many chronic diseases.

Psoriasis and metabolic syndrome, as systemic inflammatory diseases, are accompanied with higher production of ROS that react with cellular biomolecules including DNA and RNA. Other studies have proven that levels of oxidative damage to nucleic acids are increased in patients with psoriasis [12, 14, 39–42]. Kaur et al. documented that activity of antioxidant enzymes, such as superoxide dismutase and glutathione peroxidase enzymes, is reduced in patients with psoriasis [43]. Our study confirmed the results of previous studies. The level of oxidative damage to nucleic acids was significantly increased in patients with psoriasis compared to healthy controls. Subsequent analysis revealed that levels of oxidative damage to nucleic acids did not significantly differ among subjects with and without metabolic syndrome. The levels of the damage were insignificantly higher in patients with metabolic syndrome, but insignificantly lower in controls with metabolic syndrome. We do not have yet an objective explanation for the latter phenomenon. The level of oxidative damage to nucleic acids also positively correlated with the PASI score; negative correlation was documented between the PASI score and age of patients enrolled in the study.

There are many molecules that modulate inflammatory processes, and their expression is modulated by acute or chronic inflammation. One of them is ANGPTL8, a glycoprotein involved in inflammatory response, fatty acid, and saccharide metabolism. The expression of ANGPTL8 is driven by inflammatory environment, feeding, glucose, and insulin level. Its levels are elevated in patients with dyslipidemia, cardiovascular diseases, diabetes mellitus, SIRS, hepatosteatosis, hypothyroidism, diabetic retinopathy, pregnancy, PCOS (especially in patients with metabolic syndrome), and nephropathy associated with diabetes mellitus. ANGPTL8 levels negatively correlates with HDL cholesterol and LPL and positively with TCG and VEGF [44–53]. The opposite observations have been presented by Gómez-Ambrosi et al. and Wang et al. [54, 55]. Their results provided evidence that the level of ANGPTL 8 is decreased in patients with obesity and diabetes mellitus type II. These conflicting results might be explained with ANGPTL8 complexity, its involvement in a wide range of processes, or methods used for ANGPTL8 detection. Some commercial kits detect only a full-length form, while some measured both full-length and cleaved C and N-terminus of ANGPTL8. Both Gómez-Ambrosi et al. and Wang et al. used the same kit for ANGPTL8 detection.

Factors that decrease the level of ANGPTL8 includes hyperinsulinemia, TNF*α*, miRNA 143-3p from hepatocytes, and miRNA 221-3p which expression is promoted by activated macrophages in adipose cells, glucocorticoids during fasting, etc. [51, 56–58]. The level of ANGPTL is also lower in patients with Graves' disease [59]. The study conducted by Zhang et al. proved that ANGPTL8 negatively regulates NF-*κ*B activation. ANGPTL8 binds p62, as a coreceptor, and IKK*γ*, thereby forms a functional complex, and facilitates selective autophagic IKK*γ* degradation. ANGPTL 8 might suppress acute inflammatory response; it is a negative feedback regulator controlling inflammation [60]. The opposite results were described by Liao et al. The grade of intervertebral disc degeneration which is associated with inflammation positively correlated with mRNA ANGPTL8. The expression of ANGPLT8 was upregulated by TNF*α* and overexpression of ANGPTL8 significantly increased the expression of MMP3, MMP9, and IL-6 [61].

Knowing these data, we assumed that the increase or decrease of levels of ANGPTL8 in psoriatic patients and all subjects with metabolic syndrome could occur. To date, the levels of ANGPTL8 were not measured in psoriatic patients with/without metabolic syndrome. Although psoriasis as well as metabolic syndrome is related to systemic inflammatory conditions, and metabolic syndrome is associated with a higher value of ANGPTL8 in some previous studies, we found only insignificant differences among groups of patients and controls (with a lower level in controls), among patients with and without metabolic syndrome and among controls with and without metabolic syndrome. It seems that the presence of metabolic syndrome did not influence the levels of ANGPTL8; on the other hand, the presence of psoriasis slightly altered the expression of ANGPTL8. So, there is a possibility that pathological reactions involved in the pathogenesis of psoriasis might influence the expression of ANGPTL8 more than those involved in metabolic syndrome.

We might only speculate why the level of ANGPTL8 did not increase, despite the presence of inflammation and metabolic syndrome, as we expected. There are still many unknown factors and uncovered mechanisms that might interfere with the production and functions of ANGPTL8 (factors inhibiting or altering the synthesis of ANGPTL8, the interactions of ANGPTL8 with other molecules, the role of microbiota which regulates lipid metabolism and the immune system functions, and the presence of truncated variants of ANGPTL8) [62]. As we mentioned previously, the study performed by Gómez-Ambrosi et al. and Wang et al. [54, 55] did not document the increase of the ANGPTL8 level. Similar results as those obtained by Gómez-Ambrosi et al. and Wang et al. were also presented by Guo et al. and Tuhan et al. [63, 64]. They reported that the level of ANGPTL8 negatively correlated with hyperglycaemia and insulin resistance, the conditions which are normally associated with metabolic syndrome, but not with overweight or obesity. Fu et al. also pointed the conflicted results regarding the level of ANGPTL8 in human studies. They revealed the positive correlation between the level of vitamin D and ANGPTL8, so the vitamin D deficiency was associated with a lower level of ANGPTL8 [65]. The prevalence of vitamin D deficiency is high in the population of the Czech Republic as the deficiency is presented in 30-60% of Czechs [66, 67]. We cannot omit the fact that the results (level of ANGPTL8) also depend on the kit used, as mentioned earlier.

Considering these facts (unknown factors, hyperglycaemia, high intensity of insulin resistance, vitamin D deficiency, etc.), it is impossible to come to any proper conclusion why the level of ANGPTL8 did not increase. This research has raised many questions in need of further investigation.

## 5. Conclusions

The results of the study show that psoriasis significantly increased the serum level of calprotectin (indicator of systemic inflammation) and the serum level of oxidative damage to nucleic acids, however not the serum level of ANGPTL8 (indicator of systemic inflammation). The presence of MetS did not significantly affect the serum levels of calprotectin, ANGPTL8, and oxidative damage to nucleic acids in either psoriasis patients or controls. It seems that the serum level of calprotectin (but not serum level of ANGPTL8) could be used as a biomarker for monitoring the activity of psoriasis (diagnostic marker).

## Figures and Tables

**Table 1:** The levels of calprotectin, ANGPTL8, and oxidative damage to nucleic acids in controls and patients.

Parameter	Median (Q1–Q3)	Significance of the differences
Calprotectin (pg/mL)		
Controls	1943.0 (1260.0–2488.0)	*p* < 0.001
Patients	5231.0 (4159.0–8041.0)	
ANGPTL8 (ng/mL)		
Controls	11.97 (9.63–15.10)	NS
Patients	13.80 (10.83–14.72)	
Oxidative damage to nucleic acids (pg/mL)		
Controls	1730.0 (981.0–2643.4)	*p* < 0.001
Patients	3269.3 (2004.4–4505.9)	

Legend: Q1: first quartile; Q3: third quartile.

**Table 2:** The levels of calprotectin, ANGPTL8, and oxidative damage to nucleic acids in patients and controls with and without MetS.

Parameter	Q1	Median	Q3	Geometric mean	Min	Max	Significance of the differences
Calprotectin (pg/mL)							
Patients No-MetS	4298.0	4746.0	7233.8	5746.0	1976.0	12538.0	NS
Patients MetS	4020.3	5612.5	8590.8	6310.3	1360.0	12800.0	
Controls No-MetS	1185.8	1690.5	2334.0	1964.5	497.0	6299.0	NS
Controls MetS	1389.3	2232.5	2984.5	2358.7	779.0	5659.0	
ANGPTL8 (ng/mL)							
Patients No-MetS	10.78	13.80	19.00	14.70	5.78	25.50	NS
Patients MetS	10.98	13.92	19.98	14.74	4.60	24.30	
Controls No-MetS	10.02	12.20	15.09	12.63	2.30	25.40	NS
Controls MetS	9.47	11.75	15.60	12.98	2.10	32.80	
Oxidative damage to nucleic acids (pg/mL)							
Patients No-MetS	2004	2931	4506	3415	700	8150	NS
Patients MetS	2270	3517	4523	3651	500	13050	
Controls No-MetS	1250	1805	3612	2665	191	16100	NS
Controls MetS	786	1465	2339	1897	128	7800	

Legend: Q1: first quartile; Q3: third quartile.

## Data Availability

The data used to support the findings of this study are available from the corresponding author upon request.
